# Supplementary material for: Lung Ultrasound to Determine the Effect of Lower vs. Higher PEEP on Lung Aeration in Patients without ARDS—A Substudy of a Randomized Clinical Trial
Source: Diagnostics (Basel). 2023 Jun 7;13(12):1989. doi: 10.3390/diagnostics13121989 (PMC10297592; doi:10.3390/diagnostics13121989)

Figure S1. Lung ultrasound score. Each LUS image was scored with the LUS aeration score: (A) normal aeration: presence of lung sliding with A lines or ≤2 isolated B lines (0 point); (B1) moderate loss of aeration: B-lines are well-spaced and cover ≤ 50% of the pleural line (1 point); (B2) severe loss of lung aeration: B-lines cover > 50% of the pleural line (2 points); (C) consolidated lung tissue: hypoechoic or tissue–like area (3 points).

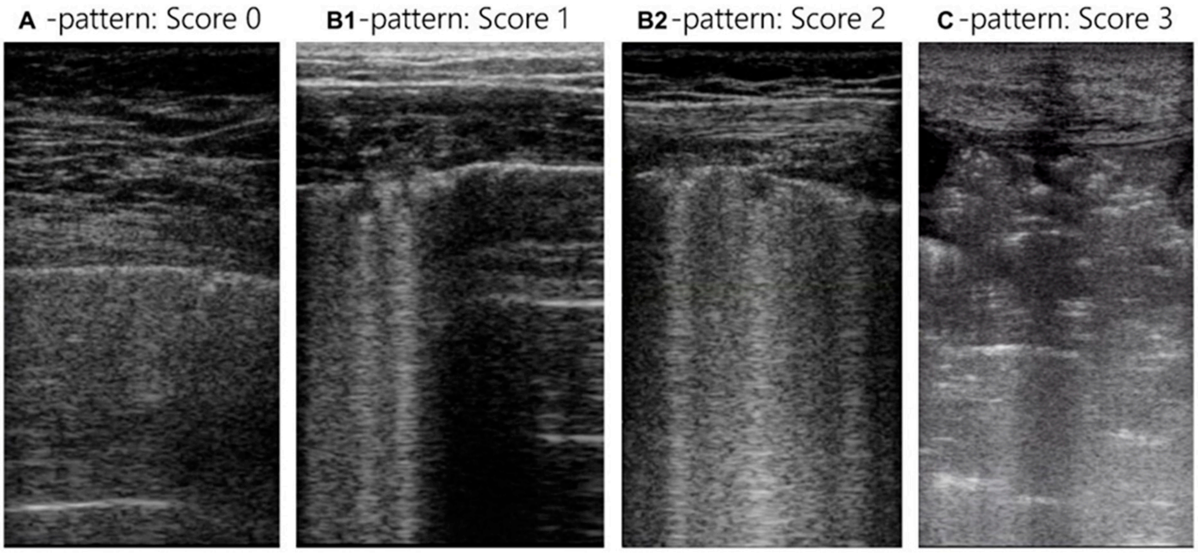

Supplement: Supplementary file 1 [file diagnostics-13-01989-s001.zip › Figure S1.pdf]
